# Global Trends and Cross-Country Differences in Authorship by Women in Academic Anaesthesiology Since 1996: A Repeated Cross-Sectional Analysis

**DOI:** 10.3390/jcm14165891

**Published:** 2025-08-21

**Authors:** Helena Schluchter, Dorothea Andel, Albert De Bettignies, Harald Andel, Eva Schaden

**Affiliations:** 1 Clinical Division of General Anaesthesia and Intensive Care Medicine, Department of Anaesthesia, Intensive Care Medicine and Pain Medicine, Medical University of Vienna, 1090 Vienna, Austria; helena.schluchter@meduniwien.ac.at (H.S.); dorothea.andel@meduniwien.ac.at (D.A.); albert.debettignies@meduniwien.ac.at (A.D.B.); eva.schaden@meduniwien.ac.at (E.S.); 2 Ludwig Boltzmann Institute Digital Health and Patient Safety, 1180 Vienna, Austria; 3 German Society of Gender-Specific Medicine, 14467 Potsdam, Germany

**Keywords:** academic anaesthesiology, authorship by women, gender equity, scientific publications, workforce diversity

## Abstract

**Background/Objectives**: Despite an increase in the number of women medical graduates, gender disparities persist in academic anaesthesiology. Women in medical science face challenges in publications, research funding, editorial board membership, and peer review, and they remain under-represented, particularly in senior authorship and leadership positions. **Methods**: This repeated cross-sectional bibliometric analysis examined global trends and cross-country differences in the representation of women as first, co-, and senior authors of peer-reviewed articles published in five high-impact anaesthesiology journals over three decades, with a focus on developments in recent years. Gender was assigned to authors’ first names algorithmically in two steps (Gender API and NamSor). **Results**: A total of 7571 publications were analysed, comprising 37,738 authors. Women constituted 11,732 (31.09%) authorships in total, and men consistently accounted for a substantial majority among authors (*p* < 0.001). Despite a significant overall increase in authorship by women—peaking in 2022 at 590 (36.88%) first authors, 2245 (37.85%) co-authors, and 402 (28.05%) senior authors (all *p* < 0.001)—stagnation was observed in recent years, with no significant changes for first and co-authors after 2016 (*p* > 0.05). Country-level analysis revealed few significant differences, with Japan consistently reporting the lowest percentages of women authors. **Conclusions**: This study underscores persistent gender disparities in academic anaesthesiology, despite a moderate increase in authorship by women over three decades. Gender disparity remains a global issue, and the recent stagnation highlights the necessity for more comprehensive efforts and innovative strategies to foster a more inclusive research community in academic anaesthesiology.

## 1. Introduction

Across scientific disciplines, including economics, social sciences, and STEM (science, technology, engineering, and mathematics) fields, women produce fewer scientific output over their careers, show higher attrition rates, and remain underrepresented in leadership [1]. EU data confirm that, despite achieving gender balance at the doctoral level in many fields, women hold only 30% of top academic posts and are less frequently authors of high-impact research or patents [2]. Workforce diversity benefits the economy, institutional performance, and individual well-being [3,4]. Innovation in science is driven by different gender, ethnic, and cultural perspectives [5]. Therefore, it is crucial to address women’s under-representation in academic leadership [6].

Despite the increasing number of women among medical graduates and physicians, gender disparity persists in higher academic positions [7,8]. The “pipeline theory” suggests that with more women in medicine, this disparity will resolve. However, equal representation remains elusive, especially among full professors and academic leaders [9]. Women medical scientists receive fewer grants and awards, and publish less frequently, which contributes to their under-representation on editorial boards, medical society chairs, and conference committees [1,10,11]. Gender disparities are also prevalent in academic anaesthesiology, even surpassing those in other medical specialties [12,13]. In U.S. academic anaesthesiology departments, women constitute 37% of faculty staff, but only 22% of professors and department chairs in 2023 [14]. Moreover, women are under-represented on editorial boards of high-impact anaesthesiology journals [15].

Academic recognition relies on the quantity of peer-reviewed publications, with authorship functioning as a measure of research productivity [16]. Collaborative research involving multi-authorship is linked to high-impact publications and more citations [17]. Junior researchers are typically first authors, while experienced investigators are listed last. The COVID-19 pandemic exacerbated gender disparities in academia by placing additional burdens on women’s research productivity due to increased caregiving and household responsibilities during widespread school and childcare closures [18,19]. In particular, women experienced a decline in first authorship on COVID-19-related research, while men’s submissions to high-impact journals and preprint servers increased [20,21].

To expand on the existing knowledge on the development of publishing activities in academic anaesthesiology [16,22,23], our study aimed to evaluate global and country-specific trends in the gender distribution of authors in five high-impact anaesthesiology journals over the past three decades with a focus on developments in recent years.

## 2. Materials and Methods

In this repeated cross-sectional study, we analysed gender differences in authorship in five high-impact anaesthesiology journals: Anaesthesia, Anesthesia and Analgesia, Anesthesiology, British Journal of Anaesthesia, and Pain. These journals were selected at the time of the study’s conceptualisation due to their high placement in journal rankings according to impact factor. To maintain consistency and comparability across study years, we maintained the same journal selection throughout the data collection process. We focused on the years 1996, 2006, and 2016 to evaluate long-term trends in authorship. In addition, to assess developments surrounding the COVID-19 pandemic, we analysed the years 2019 (pre-pandemic), and 2022 (post-pandemic). Data were collected in two phases: the first search, conducted on 25 January 2020, covered the years 1996, 2006, and 2016, and the second search, conducted on 25 December 2023, expanded the existing dataset to include the years 2019 and 2022. The bibliometric data collected included publication date, journal, and author list (first author, co-authors, senior author), including authors’ first names and affiliations.

We searched PubMed using the search strategy: (“Journal Name” [Journal]) AND (“YYYY/01/01” [Date—Publication]: “YYYY/12/31” [Date—Publication]). We included all peer-reviewed publications such as original research, reviews, editorials, and letters. For single-authored publications, the author was considered the first author. Authors’ full first names were ascertained using PubFacts (Oakland, CA, USA), ResearchGate (ResearchGate GmbH, Berlin, Germany), Scopus (Elsevier B.V., Amsterdam, The Netherlands), and Web of Science (Clarivate, Philadelphia, PA, USA). Where either the full first name or the country of affiliation could not be identified, the author was excluded from the analysis.

We determined the gender of each author based on first name and country of affiliation using two independent highly reliable algorithms [24,25]. First, we applied Gender API (https://gender-api.com, accessed on 20 October 2024; Markus Perl IT Solutions, Passau, Germany), rejecting gender-matchings with a certainty of less than 90%. Gender-matching for Asian names is known to be challenging [1]. Therefore, we reprocessed the initially excluded authors using NamSor (https://namsor.app, accessed on 21 October 2024; NAMSOR SAS, Versailles, France), which draws on a large database of Asian names. Authors with a gender-matching certainty of less than 0.9 were excluded.

### Statistical Analysis

Statistical analyses were conducted in RStudio version 2023.12.1.402 (Posit Software, PBC, Boston, MA, USA) using the tidyverse meta-package version 2.0.0 [26]. All tests were two-sided.

The association between gender and authorship type by year globally and for the ten countries with the highest number of authors (top ten) was assessed using Pearson chi-square (χ^2^) test for independence and post hoc pairwise comparisons with Bonferroni correction. Effect sizes were measured using Cramer’s V (V). Pairwise proportion tests with Bonferroni correction and Cohen’s h (h) were used to analyse cross-country differences. Fisher’s exact test was chosen for post hoc testing with odds ratio (OR, 95% confidence interval [CI]) for low expected frequencies. Logistic regression was used to assess the likelihood of a woman as a first author based on the gender of the senior author and publication year, including only publications with complete information of first and senior author. Results were presented as OR (95% CI).

Data were presented as absolute numbers and percentages, with significance set at *p* < 0.05 and *p* < 0.01 after Bonferroni correction, respectively. Effect size measures were categorised as small (V ≤ 0.10 with one degree of freedom (df); V ≤ 0.05 with four df; h ≤ 0.20), medium (V ≈ 0.30 with one df; V ≈ 0.15 with four df; h ≈ 0.50), or large (V ≥ 0.50 with one df; V ≥ 0.25 with four df; h ≥ 0.80).

## 3. Results

The PubMed search returned 7615 publications, yielding 40,887 authors in total. Due to missing information (either first name or country of affiliation) that could not be retrieved, 618 (1.51%) authors were excluded from the analysis. The remaining 40,269 (98.49%) authors underwent the gender-matching process. After excluding first name-to-gender matchings below 90% certainty for Gender API and below 0.9 certainty for NamSor, respectively, 37,738 (92.30%) authors were analysed. Supplemental Table S1 provides a comprehensive breakdown of women and men authors included for analysis, as well as excluded authors, by country and year.

### 3.1. Global Trends

There were 11,732 (31.09%) women and 26,006 (68.91%) men authors in total (*p* < 0.001; V = 0.38 with one df). This significant gender difference persisted across all analysed publication years (*p* < 0.001 for each year; V_1996_ = 0.56, V_2006_ = 0.48, V_2016_ = 0.32, V_2019_ = 0.35, V_2022_ = 0.29, each with four df), despite an increase in women authors over the last three decades (*p* < 0.001; V = 0.15 with four df), peaking at 3237 (36.11%) in 2022.

Men consistently accounted for a substantial majority among first, co-, and senior authors across all analysed publication years (Table 1). However, the share of women authors increased over time, peaking in 2022 at 590 (36.88%) first authors, 2245 (37.85%) co-authors, and 402 (28.05%) senior authors. Across multiple comparisons (1996 vs. 2006, 2006 vs. 2016, 2016 vs. 2019, and 2019 vs. 2022), there were no significant changes in the share of women as first, co-, or senior authors, except for increases in first and co-authorship between 2006 and 2016 (*p* < 0.001 for both; V = 0.10 for first authors and V = 0.09 for co-authors, each with four df) and in senior authorship between 2019 and 2022 (*p* < 0.001; V = 0.08 with four df).

### 3.2. Trends in the Top Ten Countries

The top ten countries comprised the United States of America (USA), the United Kingdom (GBR), Canada (CAN), Germany (DEU), France (FRA), Australia (AUS), Japan (JPN), The Netherlands (NLD), Denmark (DNK), and Italy (ITA). Supplemental Table S2 provides a comprehensive breakdown of women and men authors of the top ten countries. Due to missing information or unreliable gender-matching, 9.1% of Japanese authors were excluded from the analysis (Supplemental Table S1). While this is the highest exclusion rate among the top ten countries and reflects the known challenges of gender-matching Asian names, the rate was not high enough to substantially alter the findings.

**Figure 1:**
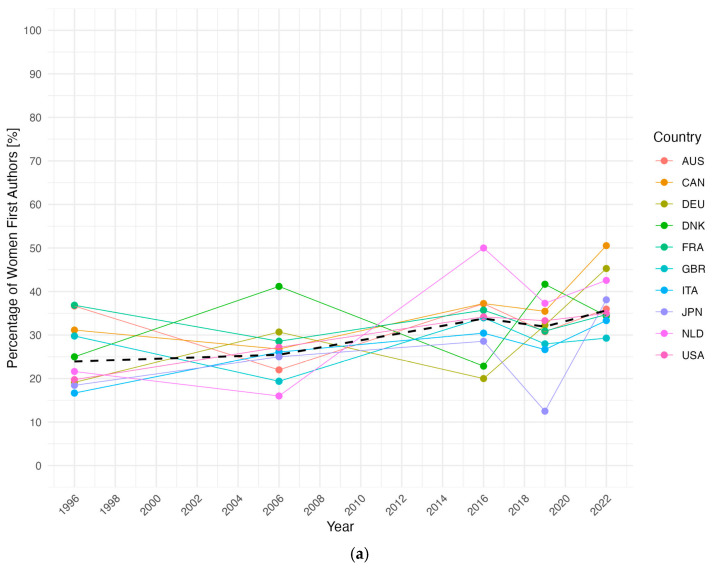

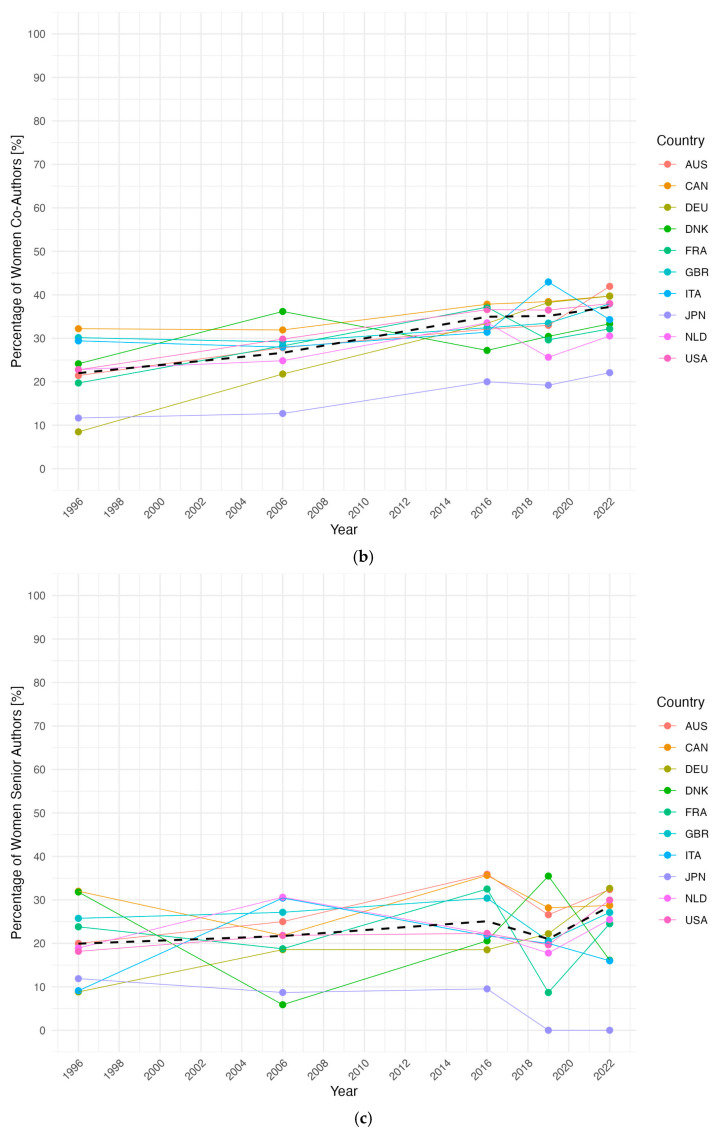
The share of women among (**a**) first, (**b**) co-, and (**c**) senior authors in the top ten countries. Country-specific percentages of women (**a**) first, (**b**) co-, and (**c**) senior authors per year, connected by solid lines. The dashed line represents the yearly mean of women (**a**) first, (**b**) co-, and (**c**) senior authors of the top ten countries. Across all authorship types, the share of women increased moderately over time but remained below gender parity in most countries, with Japan consistently showing the lowest representation. AUS, Australia; CAN, Canada; DEU, Germany; DNK, Denmark; FRA, France; GBR, the United Kingdom; ITA, Italy; JPN, Japan; NLD, The Netherlands; USA, the United States of America.

A strong predominance of men among first, co-, and senior authors persisted across all analysed publication years (*p* < 0.001 for each authorship type and year; V_min_ = 0.29 and V_max_ = 0.52 for first authors, V_min_ = 0.26 and V_max_ = 0.56 for co-authors, V_min_ = 0.44 and V_max_ = 0.60 for senior authors, each with four df). Across authorship types, the share of women increased significantly but moderately over time (*p* < 0.001; V = 0.13 with four df for first authors; *p* < 0.001; V = 0.16 with four df for co-authors; *p* < 0.001; V = 0.09 with four df for senior authors), yet it remained below gender parity in most countries (Figure 1a–c). There was one significant comparison of both first and co-authorships between consecutive years from 2006 to 2016 (*p* < 0.001; V = 0.09 with four df for first authors; *p* < 0.001; V = 0.09 with four df co-authors). In contrast, the share of women among senior authors demonstrated a notable decline between 2016 and 2019 (*p* = 0.016 with Bonferroni-corrected significance at *p* < 0.01) followed by a significant increase between 2019 and 2022 (*p* < 0.001; V = 0.08 with four df). CAN had the highest overall percentage of women authors in each category (155 first authors [37.44%]; 580 co-authors [37.57%]; 117 senior authors [29.55%]), while JPN consistently had the lowest (58 first authors [22.31%]; 121 co-authors [15.26%]; 20 senior authors [8.37%]). Notably, there were two instances of gender parity among first authors (CAN in 2022, 47 [50.54%]; NLD in 2016, 19 [50.00%]).

Pairwise proportion tests revealed a rather homogenous picture of country-specific gender distributions. While some statistically significant differences between individual countries emerged, effect sizes were generally small to moderate, indicating limited relevance and underscoring that gender disparity in authorship is a global rather than country-specific issue. The most consistent outlier was JPN, which had markedly lower representation of women compared to the other top ten countries.

### 3.3. Trends in First Authorship by Women

After excluding publications with missing information of first or senior author, 6275 out of 7571 publications (82.88%) were included in the logistic regression analysis. Model 1 assessed the likelihood of a woman as a first author based on the gender of the senior author, while model 2 included publication year as a second explanatory variable. The reference was a woman senior author (model 1) in 1996 (model 2).

Overall, the likelihood of a woman first author was significantly lower than that of a man first author (OR 0.65; 95% CI 0.58 to 0.72; *p* < 0.001). The odds of first authorship by a woman decreased significantly by about 35% with a man senior author (OR 0.65; 95% CI 0.58 to 0.73; *p* < 0.001).

This significant association persisted after including the publication year (*p* < 0.001). Model 2 showed an upward trend in women first authors, with increasing odds over time: 2006 (OR 1.17; 95% CI 0.98 to 1.41; *p* = 0.089), 2016 (OR 1.76; 95% CI 1.47 to 2.11; *p* < 0.001), 2019 (OR 1.56; 95% CI 1.32 to 1.83; *p* < 0.001). Compared to 1996, the likelihood of a woman first author increased by about 80% in 2022 (OR 1.83; 95% CI 1.55 to 2.17; *p* < 0.001).

## 4. Discussion

Despite ongoing efforts, gender parity in academic medicine remains elusive. This extensive archival study examined global trends and cross-country differences in the gender distribution of authors in high-impact anaesthesiology journals over the last three decades. A strong predominance of men authors persisted with women being particularly under-represented in senior authorship. There was a significant but moderate increase in the share of women authors overall, indicating some success of gender mainstreaming initiatives. However, this progress plateaued in recent years, suggesting that previous and current measures of promoting women have not been sufficient to sustain continuous growth. Among the top ten countries, Japan stood out as consistently having the lowest percentage of women authors. Although advances have been made, current efforts to promote gender equity in academic anaesthesiology require novel strategies for achieving sustained and meaningful progress.

While the absolute number of women authors increased substantially during the study period, the proportional growth in their representation was more modest. This indicates that part of the increase in the absolute number of women authors reflects the overall expansion in scientific output rather than a proportional improvement in women’s representation. This underscores the importance of reporting both absolute and relative measures.

Previous research has demonstrated a rise in authorship and editorial board membership of women in academic anaesthesiology, with women accounting for around 40% of total and first authors, and approximately 24% of senior authors in 2021 [16,22]. These results align with our finding of a global increase in the share of women authors over time, peaking at 590 (36.88%) first authors, 2245 (37.85%) co-authors, and 402 (28.05%) senior authors in 2022. Building on existing evidence, the extended period examined in this repeated cross-sectional study revealed a recent stagnation in the growth of first and co-authorship by women. Coupled with a U-shaped trend in the share of women senior authors since 2016, this may indicate a potential plateau in the advancement of women’s representation.

The multifactorial processes contributing to women’s under-representation in science seem to be global. Even in Canada, which had the highest percentage of women authors among the top ten countries, women accounted for less than 40% of first and co-authors and less than 30% of senior authors. In contrast, Japan was a regular outlier with the lowest proportions of women. Interestingly, while women have accounted for around 40% of the Japanese anaesthesiologist workforce since 2016 with gradual growth over the past decade [27], only 15.40% of Japanese authors in our dataset were women. On the other hand, the share of women among U.S. authors in our dataset (36.1% in 2022) exceeded the share of women in the workforce (26.1% in 2021) [28], although the proportion of women anaesthesiologists in the U.S. has also increased only marginally by a percentage point since 2015 [29]. The incremental increases in the share of women in anaesthesiology and the concentration of women in clinical practice likely contribute to the persisting gender publication gap, most pronounced in senior authorship. Further research into the mechanisms behind this finding could reveal additional strategies to support women’s academic career advancement. Although China is not among the top ten countries in our dataset, it has emerged as a rapidly growing contributor to the field of anaesthesiology research [30,31]. It will be important to monitor future developments in gender distribution, as the Chinese share of authors is likely to continue increasing.

In line with the recent stagnation in the share of women authors in our dataset, women experienced a notable decline in authorship in the early stages of the COVID-19 pandemic, especially among first, senior, and corresponding authors, while men’s submissions of COVID-19-related research to high-impact journals and preprint servers increased [20,21,32]. Existing gender disparities in academia were amplified by placing additional burdens on women’s research productivity due to increased caregiving and household responsibilities during widespread school and childcare closures [18,19]. Women often act as primary caregivers and continue to perform the main share of unpaid domestic labour, regardless of socioeconomic status and career level [8,10,33,34,35]. This limits their time for research, business travel, and networking, and restricts access to opportunities and resources in a neoliberal academic system that prioritises high-volume scientific output and international collaboration as quantitative performance metrics, while giving little regard to competing demands on time allocation [36,37]. Main drivers of the persistent gender productivity gap include shorter academic tenures and higher dropout rates among women [1]. This disparity extends to social media, where men scholars are more likely to share their own research [38,39], whereas women face disproportionate backlash and harassment online, further discouraging self-promotion and public engagement in high-profile research topics [40].

Recent declines in funding for programs focused on recruitment, internships, career development, and mentorship for women have made employees increasingly reliant on informal sponsorship [41]. However, affinity bias often leads senior staff to sponsor individuals similar to themselves, as demonstrated by our study, which identified an association between the gender of first and senior authors, in line with existing evidence [16]. While anaesthesiology shows a strong representation of women among residents and faculty, it lags in promoting women to leadership compared to other medical specialties [14]. The under-representation of women in senior positions may pose a barrier to achieving gender parity in academic anaesthesiology overall. Another concerning development is the declining institutional commitment to gender mainstreaming and diversity initiatives. Furthermore, training initiatives have remained ineffective in improving employee awareness due to a lack of structured evaluation processes [41]. Consequently, women in male-dominated fields continue to face the same issues and microaggressions that have persisted for decades, most recently demonstrated by the social media campaign #womeninmalefields [42].

Although the focus of our study was on research activity, it is important to note that gender parity in authorship is not the sole benchmark for gender equity in academic anaesthesiology. However, scientific output remains the most direct and recognised pathway to leadership positions. In contrast, clinical practice and educational contributions, which generally have a higher share of women compared to the research domain, are often undervalued in promotion criteria [13,43,44,45]. According to the “pollution theory,” fields with higher representation of women tend to be assigned lower prestige and remuneration [46]. This may contribute to the greater institutional value placed on research compared to clinical practice and education. By increasing the recognition of excellence in these domains, we could broaden access to leadership roles for women and foster inclusive academic success. Future research should also assess the gender distribution in clinical practice and medical education, which are integral to academic anaesthesiology but fall outside the scope of this study.

The stagnation in the share of women authors suggests that more comprehensive efforts are required to sustain progress. Structural changes in institutional culture and long-term commitments from leadership are crucial for achieving gender equity [13,47]. Institutions should invest in de-biasing hiring and promotion processes, supporting parents and caregivers, and implementing a rigorous evaluation framework for these efforts. Family-friendly policies, flexible work arrangements, extended research funding and grant application deadlines, as well as virtual networking and mentorship programs, including those involving men as allies, will further foster an environment where women academics can thrive [41,48,49]. The promotion of such diversity measures will benefit not only the individual, but can also improve institutional performance and scientific innovation [3,4,5].

### Limitations

Our repeated cross-sectional study analysed publications in five high-impact anaesthesiology journals from 1996, 2006, 2016, 2019, and 2022, providing insights into global trends in gender differences in authorship. However, the data represent only part of the research output by academic anaesthesiologists. The selection of anaesthesiology journals and publication years may limit the generalisability of our findings, as results may not fully capture trends in other specialty, subspecialty, and interdisciplinary publications. For instance, journals dedicated to intensive care medicine and specifically to research on COVID-19 were not included. However, our study focused on the pandemic’s impact on scientific output rather than on pandemic-related publications. Therefore, our dataset, comprising 37,738 unique authors from high-impact anaesthesiology journals, provides a robust overview of authorship trends over the past three decades.

Gender-matching algorithms assign gender based on first names and country of affiliation using public records and registration data, though accuracy can vary with cultural and contextual factors. Matching accuracy is particularly challenging for Asian names [1]. To ensure validity, we excluded gender-matchings below 90% and 0.9 certainty, respectively. Non-binary and transgender identities were not captured in our analysis and warrant further research.

Cultural and ethnic background data were not collected, and geographical allocation relied on authors’ research institutions. Future intersectional research should investigate the interplay between research output and gender, race/ethnicity, culture, religious belief, and other diversity aspects.

We did not investigate submission attempts or rejection rates, which may contribute to gender disparities due to peer-review bias [50].

This study reveals gender differences in authorship but lacks insight into underlying causes. Further research is needed to understand and address the multifactorial process for achieving gender equity.

## 5. Conclusions

Despite recognising the benefits of gender equity, women remain under-represented in academic anaesthesiology. This repeated cross-sectional study, spanning almost 30 years, demonstrated a persistent strong predominance of men, especially among senior authors. Although the share of women authors increased overall, the upward trend has stagnated more recently. Few small to moderate cross-country differences were significant, indicating that gender disparity in authorship is a global issue. Current efforts may be insufficient, and academic anaesthesiology should prioritise gender equity to benefit from a diverse research community.

## Figures and Tables

**Table 1:** Global gender distribution. Distribution of women and men among first, co-, and senior authors per year, presented in absolute numbers and percentages. Significance set at *p* < 0.05 and *p* < 0.01 after Bonferroni correction, respectively. Effect size measures defined as small (V ≤ 0.10 with one df; V ≤ 0.05 with four df), medium (V ≈ 0.30 with one df; V ≈ 0.15 with four df), and large (V ≥ 0.50 with one df; V ≥ 0.25 with four df).

Authorship	Years	Women N (%)	Men N (%)	*p* Value	Cramer’s V (df)
First Authors	All Years	2243 (31.02)	4987 (68.98)	<0.001	0.38 (1)
	1996	357 (23.71)	1149 (76.30)	<0.001	0.53 (4)
	2006	328 (26.41)	914 (73.59)	<0.001	0.47 (4)
	2016	388 (35.76)	697 (64.24)	<0.001	0.29 (4)
	2019	580 (32.28)	1217 (67.72)	<0.001	0.35 (4)
	2022	590 (36.88)	1010 (63.13)	<0.001	0.26 (4)
Co-Authors	All Years	7930 (33.20)	15,955 (66.80)	<0.001	0.34 (1)
	1996	680 (22.30)	2369 (77.70)	<0.001	0.55 (4)
	2006	971 (26.98)	2628 (73.02)	<0.001	0.46 (4)
	2016	1771 (35.66)	3195 (64.34)	<0.001	0.29 (4)
	2019	2263 (35.69)	4077 (64.31)	<0.001	0.29 (4)
	2022	2245 (37.85)	3686 (62.15)	<0.001	0.24 (4)
Senior Authors	All Years	1559 (23.54)	5064 (76.46)	<0.001	0.53 (1)
	1996	279 (20.52)	1081 (79.49)	<0.001	0.59 (4)
	2006	265 (22.75)	900 (77.25)	<0.001	0.55 (4)
	2016	271 (25.79)	780 (74.22)	<0.001	0.48 (4)
	2019	342 (21.19)	1272 (78.81)	<0.001	0.58 (4)
	2022	402 (28.05)	1031 (71.95)	<0.001	0.44 (4)

df, degree(s) of freedom; V, Cramer’s V.

## Data Availability

The data underlying this analysis were derived entirely from publicly accessible PubMed records. The search strategy is described in Section 2. The full dataset is available from the authors upon reasonable request.

## Supplementary Materials

The following supporting information can be downloaded at: https://www.mdpi.com/article/10.3390/jcm14165891/s1, Table S1. Global author inclusion and exclusion. Comprehensive breakdown of women and men authors included for analysis, as well as excluded authors, by country and year. Authors were excluded due to missing information and low gender-matching certainty (Gender API < 90%, NamSor < 0.9). Data are presented in absolute numbers and percentages. Countries are sorted alphabetically; Table S2. Top ten countries’ gender distribution. Distribution of women and men among first, co-, and senior authors by country and year. Data are presented in absolute numbers and percentages. Top ten countries are sorted by total amount of authors.

## Abbreviations

The following abbreviations are used in this manuscript:

STEM	Science, technology, engineering, and mathematics
EU	European Union
U.S.	American (i.e., of the United States of America)
COVID-19	Coronavirus disease 2019
V	Cramer’s V
h	Cohen’s h
CI	Confidence interval
*p*	*p*-value
df	Degree of freedom
USA	The United States of America
GBR	The United Kingdom
CAN	Canada
DEU	Germany
FRA	France
AUS	Australia
JPN	Japan
NLD	The Netherlands
DNK	Denmark
ITA	Italy

## References

[1] Huang J., Gates A.J., Sinatra R., Barabási A.-L. (2020). Historical Comparison of Gender Inequality in Scientific Careers across Countries and Disciplines. Proc. Natl. Acad. Sci. USA.

[2] European Commission (2025). Directorate General for Research and Innovation. She Figures 2024: Gender in Research and Innovation: Statistics and Indicators.

[3] Audette A.P., Lam S., O’Connor H., Radcliff B. (2019). (E)Quality of Life: A Cross-National Analysis of the Effect of Gender Equality on Life Satisfaction. J. Happiness Stud..

[4] European Institute for Gender Equality (EIGE) Economic Benefits of Gender Equality in the European Union. https://eige.europa.eu/newsroom/economic-benefits-gender-equality?language_content_entity=en.

[5] (2018). Science Benefits from Diversity. Nature.

[6] Astegiano J., Sebastián-González E., Castanho C.D.T. (2019). Unravelling the Gender Productivity Gap in Science: A Meta-Analytical Review. R. Soc. open sci..

[7] Eurostat Healthcare Personnel Statistics-Physicians. https://ec.europa.eu/eurostat/statistics-explained/index.php?title=Healthcare_personnel_statistics_-_physicians#Health_graduates.

[8] Jena A.B., Khullar D., Ho O., Olenski A.R., Blumenthal D.M. (2015). Sex Differences in Academic Rank in US Medical Schools in 2014. JAMA.

[9] Rochon P.A., Davidoff F., Levinson W. (2016). Women in Academic Medicine Leadership: Has Anything Changed in 25 Years?. Acad. Med..

[10] Burden M., Frank M.G., Keniston A., Chadaga S.R., Czernik Z., Echaniz M., Griffith J., Mintzer D., Munoa A., Spence J. (2015). Gender Disparities in Leadership and Scholarly Productivity of Academic Hospitalists. J. Hosp. Med..

[11] Jagsi R., Guancial E.A., Worobey C.C., Henault L.E., Chang Y., Starr R., Tarbell N.J., Hylek E.M. (2006). The “Gender Gap” in Authorship of Academic Medical Literature—A 35-Year Perspective. N. Engl. J. Med..

[12] Bissing M.A., Lange E.M.S., Davila W.F., Wong C.A., McCarthy R.J., Stock M.C., Toledo P. (2019). Status of Women in Academic Anesthesiology: A 10-Year Update. Anesth. Analg..

[13] Flexman A.M., Shillcutt S.K., Davies S., Lorello G.R. (2021). Current Status and Solutions for Gender Equity in Anaesthesia Research. Anaesthesia.

[14] AAMC Faculty Roster: U.S. Medical School Faculty..

[15] Lorello G.R., Parmar A., Flexman A.M. (2019). Representation of Women on the Editorial Board of the Canadian Journal of Anesthesia: A Retrospective Analysis from 1954 to 2018. Can. J. Anesth/J. Can. Anesth..

[16] Miller J., Chuba E., Deiner S., DeMaria S., Katz D. (2019). Trends in Authorship in Anesthesiology Journals. Anesth. Analg..

[17] Larivière V., Gingras Y., Sugimoto C.R., Tsou A. (2015). Team Size Matters: Collaboration and Scientific Impact since 1900. Asso. Info. Sci. Tech..

[18] Malisch J.L., Harris B.N., Sherrer S.M., Lewis K.A., Shepherd S.L., McCarthy P.C., Spott J.L., Karam E.P., Moustaid-Moussa N., Calarco J.M. (2020). In the Wake of COVID-19, Academia Needs New Solutions to Ensure Gender Equity. Proc. Natl. Acad. Sci. USA.

[19] Yildirim T.M., Eslen-Ziya H. (2021). The Differential Impact of COVID-19 on the Work Conditions of Women and Men Academics during the Lockdown. Gend. Work. Organ..

[20] Kibbe M.R. (2020). Consequences of the COVID-19 Pandemic on Manuscript Submissions by Women. JAMA Surg..

[21] Muric G., Lerman K., Ferrara E. (2021). Gender Disparity in the Authorship of Biomedical Research Publications During the COVID-19 Pandemic: Retrospective Observational Study. J. Med. Internet Res..

[22] Keim A.A., Pelkey M.N., Broadfoot J.E., Folley T.A., Kraus M.B., Maloney J.A., Strand N.H., Misra L. (2023). Women Authorship Trends in the Highest-Impact Anesthesiology Journals from 2005 to 2021. J. Women’s Health.

[23] Gupta N., Banerjee S., Choudhury K.J., Prabhakar H. (2021). Women Representation as First and Corresponding Authors in Neuroanesthesiology and Neurocritical Care Journals: A Retrospective Analysis. J. Neurosurg. Anesthesiol..

[24] Santamaría L., Mihaljević H. (2018). Comparison and Benchmark of Name-to-Gender Inference Services. PeerJ Comput. Sci..

[25] Sebo P. (2021). Performance of Gender Detection Tools: A Comparative Study of Name-to-Gender Inference Services. J. Med. Libr. Assoc..

[26] Wickham H., Averick M., Bryan J., Chang W., McGowan L., François R., Grolemund G., Hayes A., Henry L., Hester J. (2019). Welcome to the Tidyverse. J. Open Source Software.

[27] Japanese Ministry of Health, Labour and Welfare (2020). Statistics of Physicians, Dentists and Pharmacists.

[28] Assocation of American Medical Colleges U.S. (2022). Physician Workforce Data Dashboard—Total Physicians in Anesthesiology, 2022.

[29] Silver J.K., Ghalib R., Poorman J.A., Al-Assi D., Parangi S., Bhargava H., Shillcutt S.K. (2019). Analysis of Gender Equity in Leadership of Physician-Focused Medical Specialty Societies, 2008–2017. JAMA Intern Med..

[30] Xie G., Zhang K., Wood C., Hoeft A., Liu J., Fang X. (2016). China’s Contribution to Anesthesiology Research: A 10-Year Survey of the Literature. Anesth. Analg..

[31] Qiang W., Xiao C., Li Z., Yang L., Shen F., Zeng L., Ma P. (2022). Impactful Publications of Critical Care Medicine Research in China: A Bibliometric Analysis. Front. Med..

[32] Lerchenmüller C., Schmallenbach L., Jena A.B., Lerchenmueller M.J. (2021). Longitudinal Analyses of Gender Differences in First Authorship Publications Related to COVID-19. BMJ Open.

[33] Fox M.F., Fonseca C., Bao J. (2011). Work and Family Conflict in Academic Science: Patterns and Predictors among Women and Men in Research Universities. Soc. Stud. Sci..

[34] Jolly S., Griffith K.A., DeCastro R., Stewart A., Ubel P., Jagsi R. (2014). Gender Differences in Time Spent on Parenting and Domestic Responsibilities by High-Achieving Young Physician-Researchers. Ann. Intern. Med..

[35] OECD Stat Employment: Time Spent in Paid and Unpaid Work, by Sex. https://stats.oecd.org/index.aspx?queryid=54757.

[36] Jadidi M., Karimi F., Lietz H., Wagner C. (2018). Gender Disparities in Science?. Dropout, Productivity, Collaborations and Success of Male and Female Computer Scientists. Advs. Complex. Syst..

[37] Uhly K.M., Visser L.M., Zippel K.S. (2015). Gendered Patterns in International Research Collaborations in Academia. Stud. High. Educ..

[38] Peng H., Teplitskiy M., Romero D., Horvát E.-Á. (2022). The Gender Gap in Scholarly Self-Promotion on Social Media. arXiv.

[39] Song Y., Wang X., Li G. (2023). Can Social Media Combat Gender Inequalities in Academia? Measuring the Prevalence of the Matilda Effect in Communication. J. Comput.-Mediat. Commun..

[40] Nogrady B. (2024). Harassment of Scientists Is Surging—Institutions Aren’t Sure How to Help. Nature.

[41] LeanIn (2024). Org.

[42] Cherelus G. (2024). Is She Playing Games, or Simply Excelling in a Male Field?. The New York Times.

[43] Bosco L., Lorello G.R., Flexman A.M., Hastie M.J. (2020). Women in Anaesthesia: A Scoping Review. Br. J. Anaesth..

[44] Gisselbaek M., Barreto Chang O., Saxena S. (2023). Gender Equity in Anesthesia: Is. It Time to Rock. the Boat?. BMC Anesthesiol..

[45] Saxena S., Gisselbaek M., Berger-Estilita J., Rubulotta F. (2025). Inclusive Pathways in Anesthesiology: Addressing Structural and Cultural Barriers on International Women’s Day. Anesth. Analg..

[46] Pelley E., Carnes M. (2020). When a Specialty Becomes “Women’s Work”: Trends in and Implications of Specialty Gender Segregation in Medicine. Acad. Med..

[47] Laver K.E., Prichard I.J., Cations M., Osenk I., Govin K., Coveney J.D. (2018). A Systematic Review of Interventions to Support the Careers of Women in Academic Medicine and Other Disciplines. BMJ Open.

[48] Von Ungern-Sternberg B.S., Sommerfield A. (2021). Gender Balance in Anesthesiology: Is. a Change of Societal Mindset Needed?. Anesth. Analg..

[49] Fry R., Aragão C., Hurst K., Parker K. (2023). In a Growing Share of U.S. Marriages, Husbands and Wives Earn About the Same.

[50] Lundine J., Bourgeault I.L., Clark J., Heidari S., Balabanova D. (2018). The Gendered System of Academic Publishing. Lancet.

