# Supplementary material for: Global Trends and Cross-Country Differences in Authorship by Women in Academic Anaesthesiology Since 1996: A Repeated Cross-Sectional Analysis
Source: J Clin Med. 2025 Aug 21;14(16):5891. doi: 10.3390/jcm14165891 (PMC12387729; doi:10.3390/jcm14165891)
Supplement: Supplementary file 1 [file jcm-14-05891-s001.zip › jcm-3770622-supplementary.pdf]

**Table S1** Global author inclusion and exclusion. Comprehensive breakdown of women and men authors included for analysis, as well as excluded authors, by country and year. Authors were excluded due to missing information and low gender-matching certainty (Gender API <90%, NamSor <0.9). Data are presented in absolute numbers and percentages. Countries are sorted alphabetically.

| Country | Year | Women authors *n* (%) | Men authors *n* (%) | Excluded authors *n* (%) |
|---|---|---|---|---|
| **Albania** | **All years** | **0 (0.0)** | **1 (100.0)** | **0 (0.0)** |
| | 2022 | 0 (0.0) | 1 (100.0) | 0 (0.0) |
| **Algeria** | **All years** | **1 (50.0)** | **1 (50.0)** | **0 (0.0)** |
| | 2019 | 1 (100.0) | 0 (0.0) | 0 (0.0) |
| | 2022 | 0 (0.0) | 1 (100.0) | 0 (0.0) |
| **Argentina** | **All years** | **13 (38.3)** | **20 (58.8)** | **1 (2.9)** |
| | 2016 | 4 (28.6) | 9 (64.3) | 1 (7.1) |
| | 2019 | 2 (33.3) | 4 (66.7) | 0 (0.0) |
| | 2022 | 7 (50.0) | 7 (50.0) | 0 (0.0) |
| **Australia** | **All years** | **502 (31.8)** | **1003 (63.5)** | **74 (4.7)** |
| | 1996 | 25 (24.7) | 72 (71.3) | 4 (4.0) |
| | 2006 | 50 (24.2) | 145 (70.0) | 12 (5.8) |
| | 2016 | 73 (32.2) | 143 (63.0) | 11 (4.8) |
| | 2019 | 141 (30.1) | 308 (65.7) | 20 (4.2) |
| | 2022 | 213 (37.0) | 335 (58.3) | 27 (4.7) |
| **Austria** | **All years** | **143 (28.2)** | **351 (69.2)** | **13 (2.6)** |
| | 1996 | 25 (33.8) | 45 (60.8) | 4 (5.4) |
| | 2006 | 46 (23.0) | 152 (76.0) | 2 (1.0) |
| | 2016 | 38 (35.2) | 69 (63.9) | 1 (0.9) |
| | 2019 | 16 (29.6) | 38 (70.4) | 0 (0.0) |
| | 2022 | 18 (25.4) | 47 (66.2) | 6 (8.4) |
| **Belarus** | **All years** | **0 (0.0)** | **1 (100.0)** | **0 (0.0)** |
| | 2022 | 0 (0.0) | 1 (100.0) | 0 (0.0) |

| Country | Year | Women authors *n* (%) | Men authors *n* (%) | Excluded authors *n* (%) |
|---|---|---|---|---|
| **Belgium** | **All years** | **160 (29.7)** | **362 (67.2)** | **17 (3.1)** |
| | 1996 | 16 (19.5) | 63 (76.8) | 3 (3.7) |
| | 2006 | 30 (28.3) | 70 (66.0) | 6 (5.7) |
| | 2016 | 27 (38.6) | 39 (55.7) | 4 (5.7) |
| | 2019 | 35 (23.0) | 114 (75.0) | 3 (2.0) |
| | 2022 | 52 (40.3) | 76 (58.9) | 1 (0.8) |
| **Benin** | **All years** | **0 (0.0)** | **1 (100.0)** | **0 (0.0)** |
| | 2019 | 0 (0.0) | 1 (100.0) | 0 (0.0) |
| **Bosnia and Herzegovina** | **All years** | **0 (0.0)** | **1 (100.0)** | **0 (0.0)** |
| | 2019 | 0 (0.0) | 1 (100.0) | 0 (0.0) |
| **Brazil** | **All years** | **153 (44.0)** | **184 (52.9)** | **11 (3.1)** |
| | 1996 | 6 (50.0) | 3 (25.0) | 3 (25.0) |
| | 2006 | 22 (43.1) | 29 (56.9) | 0 (0.0) |
| | 2016 | 50 (53.8) | 40 (43.0) | 3 (3.2) |
| | 2019 | 37 (39.8) | 55 (59.1) | 1 (1.1) |
| | 2022 | 38 (38.4) | 57 (57.6) | 4 (4.0) |
| **Burkina Faso** | **All years** | **1 (100.0)** | **0 (0.0)** | **0 (0.0)** |
| | 2022 | 1 (100.0) | 0 (0.0) | 0 (0.0) |
| **Burundi** | **All years** | **0 (0.0)** | **1 (100.0)** | **0 (0.0)** |
| | 2019 | 0 (0.0) | 1 (100.0) | 0 (0.0) |
| **Cambodia** | **All years** | **0 (0.0)** | **0 (0.0)** | **1 (100.0)** |
| | 2019 | 0 (0.0) | 0 (0.0) | 1 (100.0) |
| **Canada** | **All years** | **852 (34.8)** | **1502 (61.4)** | **93 (3.8)** |
| | 1996 | 73 (30.8) | 156 (65.8) | 8 (3.4) |
| | 2006 | 72 (27.7) | 180 (69.2) | 8 (3.1) |
| | 2016 | 220 (35.6) | 368 (59.6) | 30 (4.8) |
| | 2019 | 243 (34.8) | 427 (61.2) | 28 (4.0) |
| | 2022 | 244 (38.5) | 371 (58.5) | 19 (3.0) |

| Country | Year | Women authors *n* (%) | Men authors *n* (%) | Excluded authors *n* (%) |
|---|---|---|---|---|
| **Chile** | **All years** | **14 (30.4)** | **32 (69.6)** | **0 (0.0)** |
| | 2006 | 2 (20.0) | 8 (80.0) | 0 (0.0) |
| | 2016 | 2 (20.0) | 8 (80.0) | 0 (0.0) |
| | 2019 | 1 (20.0) | 4 (80.0) | 0 (0.0) |
| | 2022 | 9 (42.9) | 12 (57.1) | 0 (0.0) |
| **China** | **All years** | **344 (25.7)** | **379 (28.3)** | **616 (46.0)** |
| | 1996 | 6 (25.0) | 8 (33.3) | 10 (41.7) |
| | 2006 | 23 (19.5) | 40 (33.9) | 55 (46.6) |
| | 2016 | 90 (25.3) | 113 (31.7) | 153 (43.0) |
| | 2019 | 100 (27.8) | 104 (29.0) | 155 (43.2) |
| | 2022 | 125 (25.9) | 114 (23.7) | 243 (50.4) |
| **Colombia** | **All years** | **7 (36.8)** | **12 (63.2)** | **0 (0.0)** |
| | 1996 | 4 (50.0) | 4 (50.0) | 0 (0.0) |
| | 2019 | 1 (16.7) | 5 (83.3) | 0 (0.0) |
| | 2022 | 2 (40.0) | 3 (60.0) | 0 (0.0) |
| **Croatia** | **All years** | **9 (69.2)** | **4 (30.8)** | **0 (0.0)** |
| | 2016 | 6 (60.0) | 4 (40.0) | 0 (0.0) |
| | 2019 | 3 (100.0) | 0 (0.0) | 0 (0.0) |
| **Cuba** | **All years** | **3 (75.0)** | **1 (25.0)** | **0 (0.0)** |
| | 2022 | 3 (75.0) | 1 (25.0) | 0 (0.0) |
| **Cyprus** | **All years** | **0 (0.0)** | **1 (100.0)** | **0 (0.0)** |
| | 2022 | 0 (0.0) | 1 (100.0) | 0 (0.0) |
| **Czech Republic** | **All years** | **29 (53.7)** | **25 (46.3)** | **0 (0.0)** |
| | 2006 | 1 (50.0) | 1 (50.0) | 0 (0.0) |
| | 2016 | 10 (47.6) | 11 (52.4) | 0 (0.0) |
| | 2019 | 9 (42.9) | 12 (57.1) | 0 (0.0) |
| | 2022 | 9 (90.0) | 1 (10.0) | 0 (0.0) |

| Country | Year | Women authors *n* (%) | Men authors *n* (%) | Excluded authors *n* (%) |
|---|---|---|---|---|
| **Denmark** | **All years** | **232 (29.0)** | **556 (69.6)** | **11 (1.4)** |
| | 1996 | 27 (25.7) | 77 (73.3) | 1 (1.0) |
| | 2006 | 25 (29.4) | 56 (65.9) | 4 (4.7) |
| | 2016 | 55 (25.1) | 161 (73.5) | 3 (1.4) |
| | 2019 | 54 (33.6) | 105 (65.2) | 2 (1.2) |
| | 2022 | 71 (31.0) | 157 (68.6) | 1 (0.4) |
| **Egypt** | **All years** | **16 (34.8)** | **30 (65.2)** | **0 (0.0)** |
| | 2006 | 4 (66.7) | 2 (33.3) | 0 (0.0) |
| | 2016 | 4 (44.4) | 5 (55.6) | 0 (0.0) |
| | 2019 | 6 (27.3) | 16 (72.7) | 0 (0.0) |
| | 2022 | 2 (22.2) | 7 (77.8) | 0 (0.0) |
| **Ethiopia** | **All years** | **5 (20.8)** | **14 (58.4)** | **5 (20.8)** |
| | 2019 | 2 (100.0) | 0 (0.0) | 0 (0.0) |
| | 2022 | 3 (13.7) | 14 (63.6) | 5 (22.7) |
| **Faroe Islands** | **All years** | **1 (100.0)** | **0 (0.0)** | **0 (0.0)** |
| | 2022 | 1 (100.0) | 0 (0.0) | 0 (0.0) |
| **Finland** | **All years** | **108 (38.0)** | **167 (58.8)** | **9 (3.2)** |
| | 1996 | 28 (28.9) | 65 (67.0) | 4 (4.1) |
| | 2006 | 27 (29.3) | 62 (67.4) | 3 (3.3) |
| | 2016 | 11 (45.8) | 11 (45.8) | 2 (8.4) |
| | 2019 | 26 (65.0) | 14 (35.0) | 0 (0.0) |
| | 2022 | 16 (51.6) | 15 (48.4) | 0 (0.0) |
| **France** | **All years** | **515 (28.3)** | **1247 (68.5)** | **59 (3.2)** |
| | 1996 | 78 (22.1) | 255 (72.0) | 21 (5.9) |
| | 2006 | 86 (26.1) | 234 (70.9) | 10 (3.0) |
| | 2016 | 122 (35.1) | 213 (61.2) | 13 (3.7) |
| | 2019 | 88 (26.3) | 239 (71.3) | 8 (2.4) |
| | 2022 | 141 (31.1) | 306 (67.4) | 7 (1.5) |

| Country | Year | Women authors *n* (%) | Men authors *n* (%) | Excluded authors *n* (%) |
|---|---|---|---|---|
| **Gambia** | **All years** | **0 (0.0)** | **2 (100.0)** | **0 (0.0)** |
| | 2022 | 0 (0.0) | 2 (100.0) | 0 (0.0) |
| **Germany** | **All years** | **624 (27.6)** | **1601 (70.9)** | **33 (1.4)** |
| | 1996 | 34 (10.7) | 279 (87.7) | 5 (1.6) |
| | 2006 | 137 (22.5) | 465 (76.2) | 8 (1.3) |
| | 2016 | 137 (29.8) | 319 (69.3) | 4 (0.9) |
| | 2019 | 174 (34.7) | 321 (63.9) | 7 (1.4) |
| | 2022 | 142 (38.6) | 217 (59.9) | 9 (2.5) |
| **Ghana** | **All years** | **1 (25.0)** | **3 (75.0)** | **0 (0.0)** |
| | 2019 | 1 (50.0) | 1 (50.0) | 0 (0.0) |
| | 2022 | 0 (0.0) | 2 (100.0) | 0 (0.0) |
| **Greece** | **All years** | **19 (35.9)** | **27 (50.9)** | **7 (13.2)** |
| | 1996 | 4 (26.7) | 11 (73.3) | 0 (0.0) |
| | 2006 | 14 (43.8) | 12 (37.5) | 6 (18.7) |
| | 2019 | 0 (0.0) | 1 (50.0) | 1 (50.0) |
| | 2022 | 1 (25.0) | 3 (75.0) | 0 (0.0) |
| **Guadeloupe** | **All years** | **1 (20.0)** | **4 (80.0)** | **0 (0.0)** |
| | 2006 | 1 (20.0) | 4 (80.0) | 0 (0.0) |
| **Honduras** | **All years** | **1 (100.0)** | **0 (0.0)** | **0 (0.0)** |
| | 2022 | 1 (100.0) | 0 (0.0) | 0 (0.0) |
| **Hong Kong** | **All years** | **20 (23.5)** | **59 (69.4)** | **6 (7.1)** |
| | 1996 | 6 (16.7) | 28 (77.8) | 2 (5.5) |
| | 2019 | 7 (28.0) | 16 (64.0) | 2 (8.0) |
| | 2022 | 7 (29.2) | 15 (62.5) | 2 (8.3) |
| **Hungary** | **All years** | **9 (26.5)** | **24 (70.6)** | **1 (2.9)** |
| | 2016 | 7 (31.8) | 14 (63.6) | 1 (4.6) |
| | 2019 | 1 (14.3) | 6 (85.7) | 0 (0.0) |
| | 2022 | 1 (20.0) | 4 (80.0) | 0 (0.0) |

| Country | Year | Women authors *n* (%) | Men authors *n* (%) | Excluded authors *n* (%) |
|---|---|---|---|---|
| **Iceland** | **All years** | **0 (0.0)** | **3 (100.0)** | **0 (0.0)** |
| | 2019 | 0 (0.0) | 1 (100.0) | 0 (0.0) |
| | 2022 | 0 (0.0) | 2 (100.0) | 0 (0.0) |
| **India** | **All years** | **65 (26.7)** | **162 (66.7)** | **16 (6.6)** |
| | 1996 | 5 (33.3) | 9 (60.0) | 1 (6.7) |
| | 2006 | 18 (21.4) | 63 (75.0) | 3 (3.6) |
| | 2016 | 11 (33.3) | 21 (63.7) | 1 (3.0) |
| | 2019 | 15 (30.6) | 30 (61.2) | 4 (8.2) |
| | 2022 | 16 (25.8) | 39 (62.9) | 7 (11.3) |
| **Indonesia** | **All years** | **1 (50.0)** | **1 (50.0)** | **0 (0.0)** |
| | 2019 | 1 (50.0) | 1 (50.0) | 0 (0.0) |
| **Iran** | **All years** | **2 (14.3)** | **11 (78.6)** | **1 (7.1)** |
| | 2006 | 1 (12.5) | 6 (75.0) | 1 (12.5) |
| | 2022 | 1 (16.7) | 5 (83.3) | 0 (0.0) |
| **Ireland** | **All years** | **77 (38.3)** | **118 (58.7)** | **6 (3.0)** |
| | 1996 | 7 (31.8) | 15 (68.2) | 0 (0.0) |
| | 2006 | 9 (21.9) | 28 (68.3) | 4 (9.8) |
| | 2016 | 21 (65.6) | 11 (34.4) | 0 (0.0) |
| | 2019 | 23 (44.2) | 28 (53.9) | 1 (1.9) |
| | 2022 | 17 (31.5) | 36 (66.7) | 1 (1.8) |
| **Israel** | **All years** | **80 (27.2)** | **198 (67.4)** | **16 (5.4)** |
| | 1996 | 8 (14.3) | 44 (78.6) | 4 (7.1) |
| | 2006 | 20 (24.7) | 60 (74.1) | 1 (1.2) |
| | 2016 | 12 (30.8) | 26 (66.7) | 1 (2.5) |
| | 2019 | 16 (27.1) | 37 (62.7) | 6 (10.2) |
| | 2022 | 24 (40.7) | 31 (52.5) | 4 (6.8) |

| Country | Year | Women authors *n* (%) | Men authors *n* (%) | Excluded authors *n* (%) |
|---|---|---|---|---|
| **Italy** | **All years** | **244 (31.5)** | **527 (67.9)** | **5 (0.6)** |
| | 1996 | 13 (22.8) | 44 (77.2) | 0 (0.0) |
| | 2006 | 39 (27.9) | 100 (71.4) | 1 (0.7) |
| | 2016 | 50 (29.4) | 117 (68.8) | 3 (1.8) |
| | 2019 | 81 (37.3) | 135 (62.2) | 1 (0.5) |
| | 2022 | 61 (31.8) | 131 (68.2) | 0 (0.0) |
| **Japan** | **All years** | **199 (14.0)** | **1093 (76.9)** | **130 (9.1)** |
| | 1996 | 59 (11.8) | 385 (76.8) | 57 (11.4) |
| | 2006 | 57 (13.0) | 340 (77.4) | 42 (9.6) |
| | 2016 | 28 (18.1) | 111 (71.6) | 16 (10.3) |
| | 2019 | 28 (14.4) | 155 (79.5) | 12 (6.1) |
| | 2022 | 27 (20.4) | 102 (77.3) | 3 (2.3) |
| **Jordan** | **All years** | **0 (0.0)** | **2 (100.0)** | **0 (0.0)** |
| | 2019 | 0 (0.0) | 2 (100.0) | 0 (0.0) |
| **Kenya** | **All years** | **5 (50.0)** | **3 (30.0)** | **2 (20.0)** |
| | 2019 | 1 (20.0) | 2 (40.0) | 2 (40.0) |
| | 2022 | 4 (80.0) | 1 (20.0) | 0 (0.0) |
| **Lebanon** | **All years** | **20 (37.7)** | **32 (60.4)** | **1 (1.9)** |
| | 1996 | 0 (0.0) | 7 (100.0) | 0 (0.0) |
| | 2006 | 7 (24.1) | 21 (72.4) | 1 (3.5) |
| | 2019 | 6 (75.0) | 2 (25.0) | 0 (0.0) |
| | 2022 | 7 (77.8) | 2 (22.2) | 0 (0.0) |
| **Libya** | **All years** | **0 (0.0)** | **1 (100.0)** | **0 (0.0)** |
| | 2022 | 0 (0.0) | 1 (100.0) | 0 (0.0) |
| **Luxembourg** | **All years** | **1 (14.3)** | **6 (85.7)** | **0 (0.0)** |
| | 2016 | 1 (16.7) | 5 (83.3) | 0 (0.0) |
| | 2019 | 0 (0.0) | 1 (100.0) | 0 (0.0) |

| Country | Year | Women authors *n* (%) | Men authors *n* (%) | Excluded authors *n* (%) |
|---|---|---|---|---|
| **Malawi** | **All years** | **2 (33.3)** | **3 (50.0)** | **1 (16.7)** |
| | 2019 | 1 (25.0) | 2 (50.0) | 1 (25.0) |
| | 2022 | 1 (50.0) | 1 (50.0) | 0 (0.0) |
| **Malaysia** | **All years** | **4 (57.1)** | **1 (14.3)** | **2 (28.6)** |
| | 2019 | 2 (40.0) | 1 (20.0) | 2 (40.0) |
| | 2022 | 2 (100.0) | 0 (0.0) | 0 (0.0) |
| **Maldives** | **All years** | **1 (100.0)** | **0 (0.0)** | **0 (0.0)** |
| | 2022 | 1 (100.0) | 0 (0.0) | 0 (0.0) |
| **Mali** | **All years** | **0 (0.0)** | **1 (14.3)** | **6 (85.7)** |
| | 2006 | 0 (0.0) | 1 (16.7) | 5 (83.3) |
| | 2022 | 0 (0.0) | 0 (0.0) | 1 (100.0) |
| **Mauritius** | **All years** | **0 (0.0)** | **1 (100.0)** | **0 (0.0)** |
| | 2022 | 0 (0.0) | 1 (100.0) | 0 (0.0) |
| **Mexico** | **All years** | **14 (28.6)** | **32 (65.3)** | **3 (6.1)** |
| | 2006 | 5 (27.8) | 13 (72.2) | 0 (0.0) |
| | 2016 | 4 (26.7) | 11 (73.3) | 0 (0.0) |
| | 2019 | 4 (28.6) | 7 (50.0) | 3 (21.4) |
| | 2022 | 1 (50.0) | 1 (50.0) | 0 (0.0) |
| **Mozambique** | **All years** | **1 (100.0)** | **0 (0.0)** | **0 (0.0)** |
| | 2019 | 1 (100.0) | 0 (0.0) | 0 (0.0) |
| **Namibia** | **All years** | **0 (0.0)** | **0 (0.0)** | **1 (100.0)** |
| | 2022 | 0 (0.0) | 0 (0.0) | 1 (100.0) |
| **Nepal** | **All years** | **2 (16.7)** | **10 (83.3)** | **0 (0.0)** |
| | 2019 | 1 (20.0) | 4 (80.0) | 0 (0.0) |
| | 2022 | 1 (14.3) | 6 (85.7) | 0 (0.0) |

| Country | Year | Women authors *n* (%) | Men authors *n* (%) | Excluded authors *n* (%) |
|---|---|---|---|---|
| **Netherlands** | **All years** | **355 (27.2)** | **912 (69.7)** | **41 (3.1)** |
| | 1996 | 41 (20.5) | 147 (73.5) | 12 (6.0) |
| | 2006 | 62 (23.1) | 194 (72.4) | 12 (4.5) |
| | 2016 | 77 (33.9) | 146 (64.3) | 4 (1.8) |
| | 2019 | 80 (26.6) | 219 (72.8) | 2 (0.6) |
| | 2022 | 95 (30.5) | 206 (66.0) | 11 (3.5) |
| **New Zealand** | **All years** | **70 (31.2)** | **148 (66.1)** | **6 (2.7)** |
| | 1996 | 4 (30.8) | 9 (69.2) | 0 (0.0) |
| | 2006 | 3 (25.0) | 9 (75.0) | 0 (0.0) |
| | 2016 | 12 (30.0) | 26 (65.0) | 2 (5.0) |
| | 2019 | 21 (29.6) | 49 (69.0) | 3 (3.4) |
| | 2022 | 30 (34.1) | 55 (62.5) | 3 (3.4) |
| **Nigeria** | **All years** | **1 (50.0)** | **1 (50.0)** | **0 (0.0)** |
| | 2022 | 1 (50.0) | 1 (50.0) | 0 (0.0) |
| **North Macedonia** | **All years** | **0 (0.0)** | **1 (100.0)** | **0 (0.0)** |
| | 2019 | 0 (0.0) | 1 (100.0) | 0 (0.0) |
| **Norway** | **All years** | **51 (26.3)** | **138 (71.1)** | **5 (2.6)** |
| | 1996 | 0 (0.0) | 2 (100.0) | 0 (0.0) |
| | 2006 | 8 (14.6) | 45 (81.8) | 2 (3.6) |
| | 2016 | 13 (38.2) | 19 (55.9) | 2 (5.9) |
| | 2019 | 17 (28.8) | 41 (69.5) | 1 (1.7) |
| | 2022 | 13 (29.6) | 31 (70.4) | 0 (0.0) |
| **Pakistan** | **All years** | **10 (43.5)** | **11 (47.8)** | **2 (8.7)** |
| | 1996 | 1 (100.0) | 0 (0.0) | 0 (0.0) |
| | 2006 | 3 (75.0) | 0 (0.0) | 1 (25.0) |
| | 2019 | 1 (25.0) | 3 (75.0) | 0 (0.0) |
| | 2022 | 5 (35.7) | 8 (57.2) | 1 (7.1) |

| Country | Year | Women authors *n* (%) | Men authors *n* (%) | Excluded authors *n* (%) |
|---|---|---|---|---|
| **Papua New Guinea** | **All years** | **1 (100.0)** | **0 (0.0)** | **0 (0.0)** |
| | 2022 | 1 (100.0) | 0 (0.0) | 0 (0.0) |
| **Poland** | **All years** | **14 (32.6)** | **29 (67.4)** | **0 (0.0)** |
| | 2016 | 10 (62.5) | 6 (37.5) | 0 (0.0) |
| | 2019 | 3 (23.1) | 10 (76.9) | 0 (0.0) |
| | 2022 | 1 (7.1) | 13 (92.9) | 0 (0.0) |
| **Portugal** | **All years** | **26 (39.4)** | **40 (60.6)** | **0 (0.0)** |
| | 1996 | 0 (0.0) | 3 (100.0) | 0 (0.0) |
| | 2016 | 9 (50.0) | 9 (50.0) | 0 (0.0) |
| | 2019 | 7 (35.0) | 13 (65.0) | 0 (0.0) |
| | 2022 | 10 (40.0) | 15 (60.0) | 0 (0.0) |
| **Puerto Rico** | **All years** | **1 (100.0)** | **0 (0.0)** | **0 (0.0)** |
| | 2019 | 1 (100.0) | 0 (0.0) | 0 (0.0) |
| **Qatar** | **All years** | **1 (25.0)** | **3 (75.0)** | **0 (0.0)** |
| | 2019 | 1 (25.0) | 3 (75.0) | 0 (0.0) |
| **Romania** | **All years** | **0 (0.0)** | **1 (100.0)** | **0 (0.0)** |
| | 2022 | 0 (0.0) | 1 (100.0) | 0 (0.0) |
| **Russia** | **All years** | **6 (42.9)** | **8 (57.1)** | **0 (0.0)** |
| | 2019 | 3 (50.0) | 3 (50.0) | 0 (0.0) |
| | 2022 | 3 (37.5) | 5 (62.5) | 0 (0.0) |
| **Rwanda** | **All years** | **0 (0.0)** | **10 (100.0)** | **0 (0.0)** |
| | 2019 | 0 (0.0) | 2 (100.0) | 0 (0.0) |
| | 2022 | 0 (0.0) | 8 (100.0) | 0 (0.0) |
| **Saudi Arabia** | **All years** | **2 (5.4)** | **34 (91.9)** | **1 (2.7)** |
| | 1996 | 0 (0.0) | 21 (95.5) | 1 (4.5) |
| | 2006 | 0 (0.0) | 3 (100.0) | 0 (0.0) |
| | 2019 | 0 (0.0) | 10 (100.0) | 0 (0.0) |
| | 2022 | 2 (100.0) | 0 (0.0) | 0 (0.0) |

| Country | Year | Women authors *n* (%) | Men authors *n* (%) | Excluded authors *n* (%) |
|---|---|---|---|---|
| **Senegal** | **All years** | **1 (100.0)** | **0 (0.0)** | **0 (0.0)** |
| | 2019 | 1 (100.0) | 0 (0.0) | 0 (0.0) |
| **Serbia** | **All years** | **4 (66.7)** | **2 (33.3)** | **0 (0.0)** |
| | 2006 | 4 (66.7) | 2 (33.3) | 0 (0.0) |
| **Sierra Leone** | **All years** | **0 (0.0)** | **1 (100.0)** | **0 (0.0)** |
| | 2019 | 0 (0.0) | 1 (100.0) | 0 (0.0) |
| **Singapore** | **All years** | **23 (26.7)** | **47 (54.7)** | **16 (18.6)** |
| | 2006 | 10 (28.6) | 18 (51.4) | 7 (20.0) |
| | 2016 | 2 (28.6) | 2 (28.6) | 3 (42.8) |
| | 2019 | 3 (15.8) | 13 (68.4) | 3 (15.8) |
| | 2022 | 8 (32.0) | 14 (56.0) | 3 (12.0) |
| **Slovakia** | **All years** | **1 (50.0)** | **1 (50.0)** | **0 (0.0)** |
| | 2019 | 1 (100.0) | 0 (0.0) | 0 (0.0) |
| | 2022 | 0 (0.0) | 1 (100.0) | 0 (0.0) |
| **Slovenia** | **All years** | **4 (33.3)** | **7 (58.3)** | **1 (8.3)** |
| | 2019 | 4 (33.3) | 7 (58.3) | 1 (8.3) |
| **South Africa** | **All years** | **56 (44.1)** | **60 (47.2)** | **11 (8.7)** |
| | 1996 | 12 (33.3) | 21 (58.3) | 3 (8.3) |
| | 2006 | 0 (0.0) | 3 (100.0) | 0 (0.0) |
| | 2016 | 0 (0.0) | 0 (0.0) | 3 (100.0) |
| | 2019 | 21 (48.8) | 20 (46.5) | 2 (4.7) |
| | 2022 | 23 (54.8) | 16 (38.1) | 3 (7.1) |
| **South Korea** | **All years** | **127 (21.0)** | **338 (56.0)** | **139 (23.0)** |
| | 1996 | 1 (5.9) | 14 (82.3) | 2 (11.8) |
| | 2006 | 12 (10.5) | 67 (58.8) | 35 (30.7) |
| | 2016 | 29 (18.6) | 96 (61.5) | 31 (19.9) |
| | 2019 | 45 (26.9) | 87 (52.1) | 35 (21.0) |
| | 2022 | 40 (26.7) | 74 (49.3) | 36 (24.0) |

| Country | Year | Women authors *n* (%) | Men authors *n* (%) | Excluded authors *n* (%) |
|---|---|---|---|---|
| **Spain** | **All years** | **204 (36.9)** | **340 (61.5)** | **9 (1.6)** |
| | 1996 | 34 (34.7) | 62 (63.3) | 2 (2.0) |
| | 2006 | 31 (35.2) | 55 (62.5) | 2 (2.3) |
| | 2016 | 53 (39.6) | 78 (58.2) | 3 (2.2) |
| | 2019 | 43 (35.9) | 76 (63.3) | 1 (0.8 |
| | 2022 | 43 (38.0) | 69 (61.1) | 1 (0.9) |
| **Sri Lanka** | **All years** | **2 (18.2)** | **7 (63.6)** | **2 (18.2)** |
| | 2022 | 2 (18.2) | 7 (63.6) | 2 (18.2) |
| **Sweden** | **All years** | **192 (32.1)** | **393 (65.6)** | **14 (2.3)** |
| | 1996 | 19 (16.3) | 90 (76.9) | 8 (6.8) |
| | 2006 | 23 (27.7) | 60 (72.3) | 0 (0.0) |
| | 2016 | 31 (41.9) | 40 (54.1) | 3 (4.0) |
| | 2019 | 48 (29.5) | 113 (69.3) | 2 (1.2) |
| | 2022 | 71 (43.8) | 90 (55.6) | 1 (0.6) |
| **Switzerland** | **All years** | **159 (21.0)** | **567 (75.0)** | **30 (4.0)** |
| | 1996 | 12 (9.1) | 108 (81.8) | 12 (9.1) |
| | 2006 | 39 (21.3) | 135 (73.8) | 9 (4.9) |
| | 2016 | 24 (18.6) | 103 (79.8) | 2 (1.6) |
| | 2019 | 43 (27.4) | 108 (68.8) | 6 (3.8) |
| | 2022 | 41 (26.5) | 113 (72.9) | 1 (0.6) |
| **Taiwan** | **All years** | **30 (9.7)** | **81 (26.1)** | **199 (64.2)** |
| | 1996 | 3 (7.2) | 20 (47.6) | 19 (45.2) |
| | 2006 | 9 (9.8) | 19 (20.6) | 64 (69.6) |
| | 2016 | 10 (14.5) | 20 (29.0) | 39 (56.5) |
| | 2019 | 5 (11.1) | 6 (13.3) | 34 (75.6) |
| | 2022 | 3 (4.8) | 16 (25.8) | 43 (69.4) |
| **Tanzania** | **All years** | **1 (9.1)** | **9 (81.8)** | **1 (9.1)** |
| | 2022 | 1 (9.1) | 9 (81.8) | 1 (9.1) |

| Country | Year | Women authors *n* (%) | Men authors *n* (%) | Excluded authors *n* (%) |
|---|---|---|---|---|
| **Thailand** | **All years** | **5 (31.3)** | **8 (50.0)** | **3 (18.7)** |
| | 1996 | 0 (0.0) | 1 (100.0) | 0 (0.0) |
| | 2006 | 1 (100.0) | 0 (0.0) | 0 (0.0) |
| | 2019 | 0 (0.0) | 2 (66.7) | 1 (33.3) |
| | 2022 | 4 (36.4) | 5 (45.4) | 2 (18.2) |
| **Turkey** | **All years** | **47 (41.6)** | **61 (54.0)** | **5 (4.4)** |
| | 1996 | 3 (33.3) | 3 (33.3) | 3 (33.3) |
| | 2006 | 30 (36.1) | 51 (61.5) | 2 (2.4) |
| | 2016 | 3 (60.0) | 2 (40.0) | 0 (0.0) |
| | 2019 | 6 (75.0) | 2 (25.0) | 0 (0.0) |
| | 2022 | 5 (62.5) | 3 (37.5) | 0 (0.0) |
| **Uganda** | **All years** | **8 (42.1)** | **11 (57.9)** | **0 (0.0)** |
| | 2019 | 2 (22.2) | 7 (77.8) | 0 (0.0) |
| | 2022 | 6 (60.0) | 4 (40.0) | 0 (0.0) |
| **Ukraine** | **All years** | **4 (44.4)** | **5 (55.6)** | **0 (0.0)** |
| | 2019 | 2 (50.0) | 2 (50.0) | 0 (0.0) |
| | 2022 | 2 (40.0) | 3 (60.0) | 0 (0.0) |
| **United Arab Emirates** | **All years** | **2 (25.0)** | **6 (75.0)** | **0 (0.0)** |
| | 2019 | 0 (0.0) | 1 (100.0) | 0 (0.0) |
| | 2022 | 2 (28.6) | 5 (71.4) | 0 (0.0) |
| **United Kingdom of Great Britain and Northern Ireland** | **All years** | **1393 (29.7)** | **3157 (67.3)** | **140 (3.0)** |
| | 1996 | 264 (27.7) | 654 (68.7) | 34 (3.6) |
| | 2006 | 132 (24.8) | 384 (72.0) | 17 (3.2) |
| | 2016 | 197 (31.3) | 412 (65.5) | 20 (3.2) |
| | 2019 | 362 (28.8) | 859 (68.5) | 34 (2.7) |
| | 2022 | 438 (33.2) | 848 (64.2) | 35 (2.6) |

| Country | Year | Women authors *n* (%) | Men authors *n* (%) | Excluded authors *n* (%) |
|---|---|---|---|---|
| **United States of America** | **All years** | **4427 (29.7)** | **9734 (65.2)** | **758 (5.1)** |
| | 1996 | 498 (20.3) | 1872 (76.4) | 80 (3.3) |
| | 2006 | 499 (26.1) | 1298 (67.8) | 117 (6.1) |
| | 2016 | 977 (32.2) | 1884 (62.2) | 170 (5.6) |
| | 2019 | 1337 (31.4) | 2705 (63.4) | 222 (5.2) |
| | 2022 | 1116 (34.2) | 1975 (60.6) | 169 (5.2) |
| **Vanuatu** | **All years** | **0 (0.0)** | **1 (100.0)** | **0 (0.0)** |
| | 2022 | 0 (0.0) | 1 (100.0) | 0 (0.0) |
| **Viet Nam** | **All years** | **2 (22.2)** | **7 (77.8)** | **0 (0.0)** |
| | 2006 | 2 (22.2) | 7 (77.8) | 0 (0.0) |
| **Zambia** | **All years** | **1 (25.0)** | **2 (50.0)** | **1 (25.0)** |
| | 2019 | 0 (0.0) | 1 (50.0) | 1 (50.0) |
| | 2022 | 1 (50.0) | 1 (50.0) | 0 (0.0) |
| **Zimbabwe** | **All years** | **0 (0.0)** | **4 (100.0)** | **0 (0.0)** |
| | 1996 | 0 (0.0) | 2 (100.0) | 0 (0.0) |
| | 2019 | 0 (0.0) | 1 (100.0) | 0 (0.0) |
| | 2022 | 0 (0.0) | 1 (100.0) | 0 (0.0) |

**Table S2** Top ten countries' gender distribution. Distribution of women and men among first, co-, and senior authors by country and year. Data are presented in absolute numbers and percentages. Top ten countries are sorted by total amount of authors.

| Country | Authorship | Year | Women *n* (%) | Men *n* (%) |
|---|---|---|---|---|
| **USA** | **First Authors** | **All Years** | **801 (30.0)** | **1865 (70.0)** |
| | | **1996** | 113 (19.8) | 459 (80.2) |
| | | **2006** | 100 (27.2) | 268 (72.8) |
| | | **2016** | 147 (34.2) | 283 (65.8) |
| | | **2019** | 243 (33.3) | 487 (66.7) |
| | | **2022** | 198 (35.0) | 368 (65.0) |
| | **Co-Authors** | **All Years** | **3076 (34.1)** | **5945 (65.9)** |
| | | **1996** | 290 (22.7) | 985 (77.3) |
| | | **2006** | 325 (29.8) | 765 (70.2) |
| | | **2016** | 737 (36.6) | 1277 (63.4) |
| | | **2019** | 960 (36.5) | 1671 (63.5) |
| | | **2022** | 764 (38.0) | 1247 (62.0) |
| | **Senior Authors** | **All Years** | **550 (22.2)** | **1924 (77.8)** |
| | | **1996** | 95 (18.2) | 428 (81.8) |
| | | **2006** | 74 (21.8) | 265 (78.2) |
| | | **2016** | 93 (22.3) | 324 (77.7) |
| | | **2019** | 134 (19.7) | 547 (80.3) |
| | | **2022** | 154 (30.0) | 360 (70.0) |
| **GBR** | **First Authors** | **All Years** | **322 (28.1)** | **822 (71.9)** |
| | | **1996** | 95 (29.8) | 224 (70.2) |
| | | **2006** | 31 (19.4) | 129 (80.6) |
| | | **2016** | 36 (34.0) | 70 (66.0) |
| | | **2019** | 78 (28.0) | 201 (72.0) |
| | | **2022** | 82 (29.3) | 198 (70.7) |
| | **Co-Authors** | **All Years** | **820 (33.9)** | **1601 (66.1)** |
| | | **1996** | 101 (30.1) | 234 (69.9) |

| | | | | |
|---|---|---|---|---|
| | | **2006** | 63 (29.2) | 153 (70.8) |
| | | **2016** | 130 (32.4) | 271 (67.6) |
| | | **2019** | 234 (33.5) | 465 (66.5) |
| | | **2022** | 292 (37.9) | 478 (62.1) |
| | **Senior Authors** | **All Years** | **251 (25.5)** | **734 (74.5)** |
| | | **1996** | 68 (25.8) | 196 (74.2) |
| | | **2006** | 38 (27.1) | 102 (72.9) |
| | | **2016** | 31 (30.4) | 71 (69.6) |
| | | **2019** | 50 (20.6) | 193 (79.4) |
| | | **2022** | 64 (27.1) | 172 (72.9) |
| **Country** | **Authorship** | **Year** | **Women *n* (%)** | **Men *n* (%)** |
| **CAN** | **First Authors** | **All Years** | **155 (37.4)** | **259 (62.6)** |
| | | **1996** | 19 (31.1) | 42 (68.9) |
| | | **2006** | 15 (26.8) | 41 (73.2) |
| | | **2016** | 35 (37.2) | 59 (62.8) |
| | | **2019** | 39 (35.5) | 71 (64.5) |
| | | **2022** | 47 (50.5) | 46 (49.5) |
| | **Co-Authors** | **All Years** | **580 (37.6)** | **964 (62.4)** |
| | | **1996** | 38 (32.2) | 80 (67.8) |
| | | **2006** | 45 (31.9) | 96 (68.1) |
| | | **2016** | 154 (37.8) | 253 (62.2) |
| | | **2019** | 173 (38.4) | 277 (61.6) |
| | | **2022** | 170 (39.7) | 258 (60.3) |
| | **Senior Authors** | **All Years** | **117 (29.5)** | **279 (70.5)** |
| | | **1996** | 16 (32.0) | 34 (68.0) |
| | | **2006** | 12 (21.8) | 43 (78.2) |
| | | **2016** | 31 (35.6) | 56 (64.4) |
| | | **2019** | 31 (28.8) | 79 (71.8) |
| | | **2022** | 27 (28.7) | 67 (71.3) |
| **DEU** | **First Authors** | **All Years** | **99 (29.2)** | **240 (70.8)** |

| | | | | |
|---|---|---|---|---|
| | | **1996** | 13 (19.1) | 55 (80.9) |
| | | **2006** | 31 (30.7) | 70 (69.3) |
| | | **2016** | 11 (20.0) | 44 (80.0) |
| | | **2019** | 20 (32.3) | 42 (67.7) |
| | | **2022** | 24 (45.3) | 29 (54.7) |
| | **Co-Authors** | **All Years** | **459 (29.7)** | **1087 (70.3)** |
| | | **1996** | 15 (8.5) | 162 (91.5) |
| | | **2006** | 88 (21.8) | 316 (78.2) |
| | | **2016** | 116 (33.4) | 231 (66.6) |
| | | **2019** | 138 (38.2) | 223 (61.8) |
| | | **2022** | 102 (39.7) | 155 (60.3) |
| | **Senior Authors** | **All Years** | **66 (19.4)** | **274 (80.6)** |
| | | **1996** | 6 (8.8) | 62 (91.2) |
| | | **2006** | 18 (18.6) | 79 (81.4) |
| | | **2016** | 10 (18.5) | 44 (81.5) |
| | | **2019** | 16 (22.2) | 56 (77.8) |
| | | **2022** | 16 (32.7) | 33 (67.3) |
| **FRA** | **First Authors** | **All Years** | **84 (33.3)** | **168 (66.7)** |
| | | **1996** | 21 (36.8) | 36 (63.2) |
| | | **2006** | 14 (28.6) | 35 (71.4) |
| | | **2016** | 15 (35.7) | 27 (64.3) |
| | | **2019** | 17 (30.9) | 38 (69.1) |
| | | **2022** | 17 (34.7) | 32 (65.3) |
| **Country** | **Authorship** | **Year** | **Women *n* (%)** | **Men *n* (%)** |
| | **Co-Authors** | **All Years** | **377 (29.9)** | **883 (70.1)** |
| | | **1996** | 42 (19.7) | 171 (80.3) |
| | | **2006** | 63 (28.3) | 160 (71.7) |
| | | **2016** | 94 (37.2) | 159 (62.8) |
| | | **2019** | 67 (29.6) | 159 (70.4) |
| | | **2022** | 111 (32.2) | 234 (67.8) |

| | | | | |
|---|---|---|---|---|
| | **Senior Authors** | **All Years** | **54 (21.6)** | **196 (78.4)** |
| | | **1996** | 15 (23.8) | 48 (76.2) |
| | | **2006** | 9 (18.8) | 39 (81.2) |
| | | **2016** | 13 (32.5) | 27 (67.5) |
| | | **2019** | 4 (8.7) | 42 (91.3) |
| | | **2022** | 13 (24.5) | 40 (75.5) |
| **AUS** | **First Authors** | **All Years** | **107 (32.6)** | **221 (67.4)** |
| | | **1996** | 11 (36.7) | 19 (63.3) |
| | | **2006** | 11 (22.0) | 39 (78.0) |
| | | **2016** | 16 (37.2) | 27 (62.8) |
| | | **2019** | 28 (30.8) | 63 (69.2) |
| | | **2022** | 41 (36.0) | 73 (64.0) |
| | **Co-Authors** | **All Years** | **309 (35.1)** | **572 (64.9)** |
| | | **1996** | 9 (21.4) | 33 (78.6) |
| | | **2006** | 27 (27.8) | 70 (72.2) |
| | | **2016** | 43 (32.1) | 91 (67.9) |
| | | **2019** | 92 (33.0) | 187 (67.0) |
| | | **2022** | 138 (41.9) | 191 (58.1) |
| | **Senior Authors** | **All Years** | **86 (29.1)** | **210 (70.9)** |
| | | **1996** | 5 (20.0) | 20 (80.0) |
| | | **2006** | 12 (25.0) | 36 (75.0) |
| | | **2016** | 14 (35.9) | 25 (64.1) |
| | | **2019** | 21 (26.6) | 58 (73.4) |
| | | **2022** | 34 (32.4) | 71 (67.6) |
| **JPN** | **First Authors** | **All Years** | **58 (22.3)** | **202 (77.7)** |
| | | **1996** | 19 (18.4) | 84 (81.6) |
| | | **2006** | 19 (25.0) | 57 (75.0) |
| | | **2016** | 8 (28.6) | 20 (71.4) |
| | | **2019** | 4 (12.5) | 28 (87.5) |
| | | **2022** | 8 (38.1) | 13 (61.9) |

| | | | | |
|---|---|---|---|---|
| | **Co-Authors** | **All Years** | **121 (15.3)** | **672 (84.7)** |
| | | **1996** | 28 (11.7) | 212 (88.3) |
| | | **2006** | 32 (12.7) | 220 (87.3) |
| | | **2016** | 18 (20.0) | 72 (80.0) |
| | | **2019** | 24 (19.2) | 101 (80.8) |
| | | **2022** | 19 (22.1) | 67 (77.9) |

| Country | Authorship | Year | Women *n* (%) | Men *n* (%) |
|---|---|---|---|---|
| | **Senior Authors** | **All Years** | **20 (8.4)** | **219 (91.6)** |
| | | **1996** | 12 (11.9) | 89 (88.1) |
| | | **2006** | 6 (8.7) | 63 (91.3) |
| | | **2016** | 2 (9.5) | 19 (90.5) |
| | | **2019** | 0 (0.0) | 26 (100.0) |
| | | **2022** | 0 (0.0) | 22 (100.0) |
| **NLD** | **First Authors** | **All Years** | **77 (33.3)** | **154 (66.7)** |
| | | **1996** | 8 (21.6) | 29 (78.4) |
| | | **2006** | 8 (16.0) | 42 (84.0) |
| | | **2016** | 19 (50.0) | 19 (50.0) |
| | | **2019** | 22 (37.3) | 37 (62.7) |
| | | **2022** | 20 (42.6) | 27 (57.4) |
| | **Co-Authors** | **All Years** | **227 (27.8)** | **591 (72.2)** |
| | | **1996** | 26 (22.8) | 88 (77.2) |
| | | **2006** | 39 (24.8) | 118 (75.2) |
| | | **2016** | 50 (33.6) | 99 (66.4) |
| | | **2019** | 50 (25.6) | 145 (74.4) |
| | | **2022** | 62 (30.5) | 141 (69.5) |
| | **Senior Authors** | **All Years** | **51 (23.4)** | **167 (76.6)** |
| | | **1996** | 7 (18.9) | 30 (81.1) |
| | | **2006** | 15 (30.6) | 34 (69.4) |
| | | **2016** | 8 (22.2) | 28 (77.8) |
| | | **2019** | 8 (17.8) | 37 (82.2) |

| | | | | |
|---|---|---|---|---|
| | | **2022** | 13 (25.5) | 38 (74.5) |
| **DNK** | **First Authors** | **All Years** | **47 (32.6)** | **97 (67.4)** |
| | | **1996** | 6 (25.0) | 18 (75.0) |
| | | **2006** | 7 (41.2) | 10 (58.8) |
| | | **2016** | 8 (22.9) | 27 (77.1) |
| | | **2019** | 15 (41.7) | 21 (58.3) |
| | | **2022** | 11 (34.4) | 21 (65.6) |
| | **Co-Authors** | **All Years** | **154 (30.3)** | **355 (69.7)** |
| | | **1996** | 14 (24.1) | 44 (75.9) |
| | | **2006** | 17 (36.2) | 30 (63.8) |
| | | **2016** | 40 (27.2) | 107 (72.8) |
| | | **2019** | 28 (30.4) | 64 (69.6) |
| | | **2022** | 55 (33.3) | 110 (66.7) |
| | **Senior Authors** | **All Years** | **31 (23.0)** | **104 (77.0)** |
| | | **1996** | 7 (31.8) | 15 (68.2) |
| | | **2006** | 1 (5.9) | 16 (94.1) |
| | | **2016** | 7 (20.6) | 27 (79.4) |
| | | **2019** | 11 (35.5) | 20 (64.5) |
| | | **2022** | 5 (16.1) | 26 (83.9) |

| Country | Authorship | Year | Women *n* (%) | Men *n* (%) |
|---|---|---|---|---|
| **ITA** | **First Authors** | **All Years** | **32 (27.8)** | **83 (72.2)** |
| | | **1996** | 2 (16.7) | 10 (83.3) |
| | | **2006** | 6 (26.1) | 17 (73.9) |
| | | **2016** | 7 (30.4) | 16 (69.6) |
| | | **2019** | 8 (26.7) | 22 (73.3) |
| | | **2022** | 9 (33.3) | 18 (66.7) |
| | **Co-Authors** | **All Years** | **189 (34.7)** | **355 (65.3)** |
| | | **1996** | 10 (29.4) | 24 (70.6) |
| | | **2006** | 26 (28.0) | 67 (72.0) |
| | | **2016** | 38 (31.4) | 83 (68.6) |

| | | | |
|---|---|---|---|
| | **2019** | 67 (42.9) | 89 (57.1) |
| | **2022** | 48 (34.3) | 92 (65.7) |
| **Senior Authors** | **All Years** | **23 (20.5)** | **89 (79.5)** |
| | **1996** | 1 (9.1) | 10 (90.9) |
| | **2006** | 7 (30.4) | 16 (69.6) |
| | **2016** | 5 (21.7) | 18 (78.3) |
| | **2019** | 6 (20.0) | 24 (80.0) |
| | **2022** | 4 (16.0) | 21 (84.0) |

*AUS, Australia; CAN, Canada; DEU, Germany; DNK, Denmark; FRA, France; GBR, the United Kingdom; ITA, Italy; JPN, Japan; NLD, the Netherlands; USA, the United States of America.*
